# Inhibition of miR-542-3p augments autophagy to promote diabetic corneal wound healing

**DOI:** 10.1186/s40662-023-00370-1

**Published:** 2024-01-03

**Authors:** Danling Liao, Shijia Wei, Jianzhang Hu

**Affiliations:** Department of Ophthalmology, Fujian Medical University Union Hospital, 29 Xinquan Road, Fuzhou, 350005 China

**Keywords:** Diabetic corneal neuropathy, Autophagy, Nerve regeneration, miR-542-3p, ATG4D

## Abstract

**Background:**

Autophagy has recently been shown to be critical for protecting peripheral nerve regeneration. This study explored the impact of miR-542-3p on diabetic corneal nerve regeneration and epithelial healing through the regulation of autophagy.

**Methods:**

A type 1 diabetes model was established in male mice through streptozotocin administration. Immunofluorescence staining of β-Tubulin III and sodium fluorescein staining were performed to observe corneal nerve fiber density and corneal epithelial healing, respectively. Western blotting, immunofluorescence and transmission electron microscopy were used to determine autophagy levels. Subconjunctival injection of RAPA and 3-MA altered autophagy levels; with them, we evaluated the role of autophagy in diabetic keratopathy. miRNA sequencing and bioinformatics analysis were performed to identify miRNA-mRNA networks with potential autophagy-regulating roles, and miR-542-3p was measured by quantitative real-time polymerase chain reaction (qRT-PCR). miR-542-3p antagomir was injected subconjunctivally to assess the role in diabetic corneal neuropathy.

**Results:**

Our data suggest that autophagy is suppressed in the diabetic corneal nerve and that activation of autophagy promotes diabetic corneal wound healing. We identified a potential autophagy-regulating miRNA-mRNA network in the diabetic trigeminal ganglion, in which miR-542-3p expression was significantly upregulated. Inhibition of miR-542-3p significantly enhanced the level of autophagy in trigeminal ganglion by upregulating ATG4D expression, thereby accelerating diabetic corneal nerve regeneration and epithelial healing.

**Conclusions:**

Dysregulated autophagy is an important contributor to delayed diabetic corneal injury healing. Inhibiting miR-542-3p promotes diabetic corneal nerve regeneration and epithelial healing through autophagy activation by ATG4D.

**Supplementary Information:**

The online version contains supplementary material available at 10.1186/s40662-023-00370-1.

## Background

Diabetic keratopathy (DK) is a common ocular complication of diabetes that seriously threatens the vision of diabetic patients. DK results in decreased corneal sensitivity, persistent epithelial defects, and delayed epithelial healing [1, 2]. Corneal neuropathy occurs in the early stages of diabetic peripheral neuropathy and is characterized by decreased nerve density and nerve fiber length, increased nerve tortuosity, leading to corneal sensory dysfunction [3]. The neuropeptides secreted by nerve endings are reduced concurrently, leading to corneal trophic dysfunction [3, 4]. Currently, abnormalities in the structure and function of corneal nerves are recognized as important causes of DK [5]. Most of the nerve fibers in the cornea originate from the trigeminal ganglion (TG) [1]. Corneal nerve fibers carry sensory signals, nourish the cornea, and maintain its normal form and function [6, 7]. The neurological distribution of corneal nerves greatly affects corneal epithelial cells. Furthermore, neurotrophic factors, growth factors, and neuropeptides secreted by corneal nerve fibers stimulate corneal epithelial healing [8]. The mechanism of diabetic corneal neuropathy remains unclear. Thus, it is theoretically and clinically important to investigate the mechanism of diabetic corneal neuropathy to identify new molecular therapeutic targets for diagnosing and treating this condition.

Autophagy is a highly conserved process of protein or organelle degradation in eukaryotes that enables the removal of abnormally folded proteins and damaged or redundant organelles through lysosomes [9]. It leads to the renewal of cell metabolism and organelles, thereby maintaining cell survival, differentiation and the homeostasis of the intracellular environment [10]. Dysregulated autophagy is involved in the development of various diseases [9–12], including diabetic peripheral neuropathy and other complications of diabetes [13–15]. Research has shown that autophagy contributes to nerve tissue structural plasticity, which reduces scar formation and facilitates nerve repair after injury [16, 17]. The moderated activation of autophagy reveals a protective effect on nerve cells [18–20]. Recent studies have demonstrated that modulating the autophagy-related genes Atg4B and Atg5 in diabetic mice promotes the regeneration of corneal nerves [21, 22]. However, comprehensive details regarding the regulatory mechanism are lacking, and further investigation into the mechanisms involved in regulating autophagy is needed to identify therapeutic targets for diabetic corneal neuropathy.

miRNAs are a class of widely distributed endogenous single-stranded noncoding RNAs [23, 24]. They function by attaching to the 3'UTR of target mRNAs, forming an RNA-induced silencing complex to degrade target mRNAs or reduce targeted mRNA translation efficiency [23]. miRNAs have a critical function in diabetes and chronic neurological diseases by regulating various physiological processes, including autophagy [25–27]. Abnormal expression of miR-182, miR-34c, miR-181a-5p, and miR-223-5p in DK is involved in corneal injury healing [21, 22, 28, 29]. Most of the current research on miRNAs in DK has focused on a single miRNA. Therefore, there is a substantial gap in knowledge regarding the role of miRNAs in diabetic corneal neuropathy. Screening miRNAs within the whole transcriptome provides a more solid understanding of the biological process of diabetic corneal neuropathy, which should aid in developing promising mimics or inhibitors targeting miRNAs for treating DK [30, 31].

In this study, we investigated the role of autophagy in diabetic corneal injury healing and explored a new targeted miRNA that regulates autophagy and the corresponding mechanism through high-throughput sequencing of the TG tissue in diabetic and normal mice. The aim of the study is to provide potential diagnostic biomarkers for diabetic corneal neuropathy as well as possible treatment options for diabetic corneal wound healing.

## Methods

### Experimental animals

We purchased C57BL/6 J male mice (6–8 weeks old) from Beijing SPF Biotechnology Co., Ltd. (Beijing, China). The Guidelines for the Care and Use of Laboratory Animals and the Principles for the Utilization and Care of Vertebrate Animals were strictly followed. The Institutional Animal Care and Use Committee of Fujian Medical University approved all animal experiments (IACUC FJMU 2021-0454). The procedures conformed to the guidelines of the Association for Research and Vision and Ophthalmology statement for the use of animals in ophthalmic and vision research. Streptozotocin (STZ; Sigma-Aldrich, USA) mixed with citrate-citric acid buffer was prepared for induction of type 1 diabetes mellitus (T1DM). Experimental mice (n = 65) were injected intraperitoneally with low-dose STZ (50 mg/kg) for five days, and control mice (n = 45) were injected intraperitoneally with an equal amount of citrate-citric acid buffer. Before intraperitoneal injection, we measured baseline blood glucose levels in both groups of mice, and then random intravenous blood glucose concentrations were measured every four weeks until 16 weeks. Both groups of mice were given a normal diet. Experimental mice whose blood glucose level exceeded 16.7 mmol/L in each measurement were considered to be diabetic.

### Corneal epithelial debridement healing

**Fig. 1:**
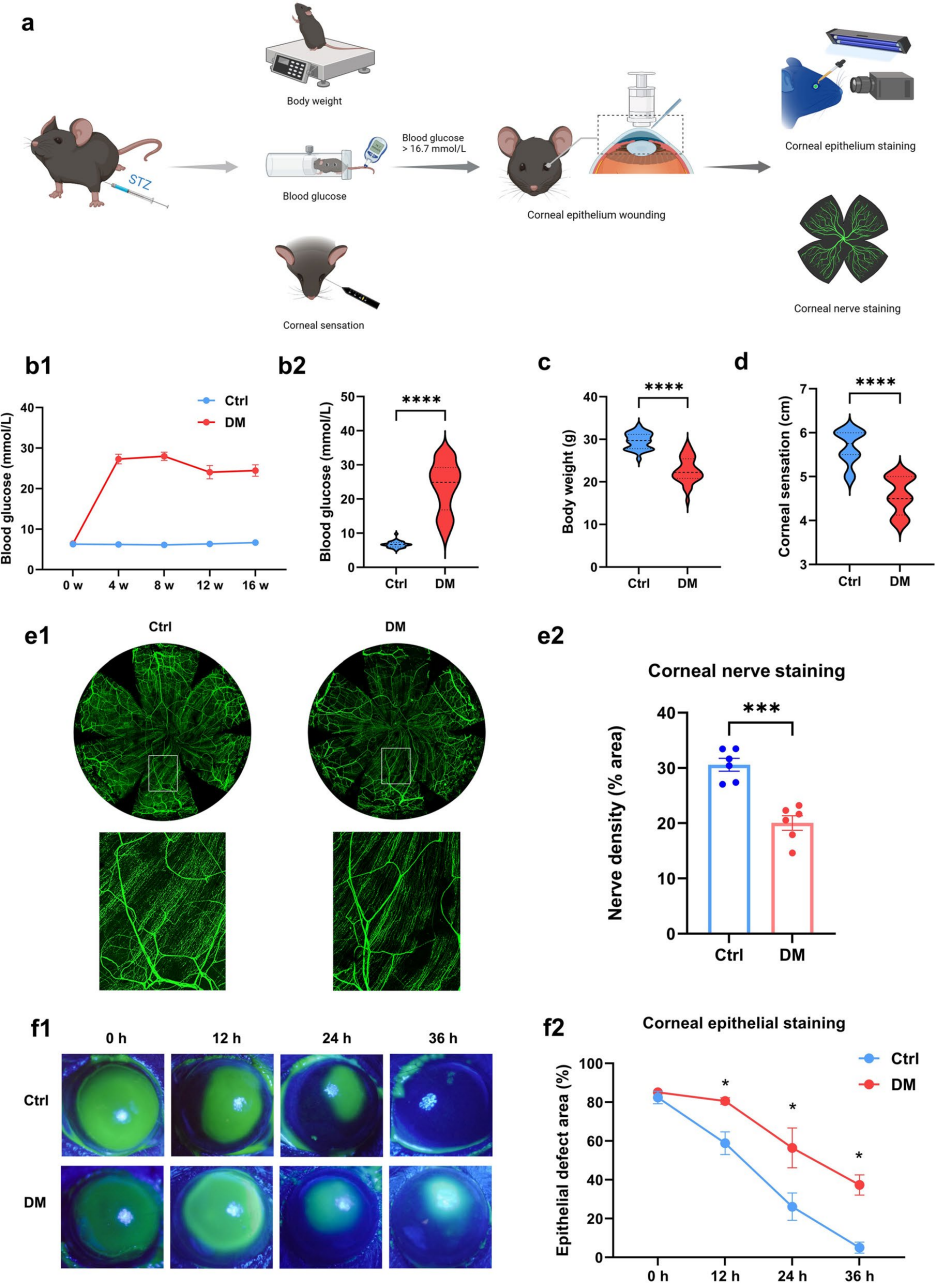
Sustained hyperglycemia delayed corneal debridement healing in mice. **a** Flowchart for the establishment of type 1 diabetes mellitus (T1DM) mice and corneal debridement healing. **b** Blood glucose values of normal (Ctrl) and diabetic (DM) mice. **b1** Blood glucose values at 0, 4, 8, 12, 16 weeks after intraperitoneal injection. **b2** Significant differences in blood glucose levels between the two groups at 16 weeks. **c** Body weight values of Ctrl and DM mice at 16 weeks. **d** Corneal sensitivity of Ctrl and DM mice at 16 weeks. **e** Corneal nerves whole-mount staining on day 5 of Ctrl and DM mice after debridement (n = 6 per group). **e1** Fluorescent images of corneal nerve staining. **e2** Peripheral corneal nerve density. **f** Corneas stained with fluorescein sodium at 0, 12, 24, and 36 h in Ctrl and DM mice after debridement (n = 6 per group). **f1** Fluorescein-stained images of corneas. **f2** Percentage of epithelial defect area. *, *P* < 0.05; ***, *P* < 0.001; ****, *P* < 0.0001

An intraperitoneal injection of 1.25% tribromoethanol (0.2 mL/10 g) was used to induce general anesthesia in experimental mice, followed by local anesthesia of the ocular surface with 0.5% promethazine hydrochloride. An AlgerBrush II ring drill (Alger Inc., USA) was applied to exfoliate the corneal epithelium (2.5 mm), and then ofloxacin eye ointment was applied to prevent infection (Fig. 1a). At 0, 12, 24 and 36 h after debridement, experimental mice were grasped to briefly expose the eyeballs after local anesthesia, the defect area was observed with 0.25% sodium fluorescein staining and photographed under a microscope with cobalt-blue light. Image J was used to calculate the ratio of fluorescent stained area to corneal area as the percentage of epithelial defect area. At least three mice in each group were used for separate independent experiments.

### Corneal sensitivity measurement

The Cochet-Bonnet esthesiometer (Luneau ophthalmology, France) was used to estimate corneal sensitivity in this study. On day 5 after corneal epithelial debridement, each eye of unanesthetized mice was measured at least three times, and the blink reflex was judged as a positive response. Each measurement was shortened by 0.5 cm until a positive response was observed. The corneal sensitivity threshold is the longest filament length that results in a positive response. At least three mice in each group were used for separate independent experiments.

### Whole-mount staining of corneal nerves

Experimental mice were sacrificed and fresh intact corneal tissue was harvested and fixed in Zamboni fixative on day 5 after corneal epithelial debridement. Corneal tissue was subsequently blocked and permeabilized in a solution containing 0.3% Triton X-100 and 10% goat serum. Anti-β-tubulin III antibody (657404, Biolegend, USA) was used for overnight staining. Corneal tissue was scanned under a laser confocal microscope (Leica, Germany). At least three mice in each group were used for separate independent experiments.

### Western blot assay

The TG tissues were harvested on day 5 after corneal epithelial debridement and lysed with RIPA reagent (Beyotime, Shanghai, China) supplemented with 1% Phenylmethylsulfonyl Fluoride (PMSF) reagent (Beyotime). Protein samples were electrophoresed on Sodium dodecyl sulfate–polyacrylamide gels, transferred to polyvinylidene fluoride membranes, and blocked with skim milk. Afterward, the membranes were incubated in solutions of the corresponding primary antibodies and horseradish peroxidase-conjugated secondary antibodies. Eventually, Enhanced chemiluminescence was used to visualize proteins bands. At least three independent experiments were performed. The primary antibodies used for the study were as follows: anti-p62 antibody (p0067; Sigma-Aldrich, USA), anti-LC3B (ab192890; Abcam, USA), anti-ATG4D (ab237751; Abcam, USA), and anti-β-actin (ab8226; Abcam, USA).

### Subconjunctival injection

After general and ocular surface anesthesia of mice, 5 µL of solution was injected into the lower bulbar conjunctiva with a microsyringe. For the injection of two solutions, different solutions were injected into the upper and lower bulbar conjunctiva. At least three mice in each group were used for separate independent experiments. All solutions were injected 24 h before, 0 h, and 24 h after debridement. The solutions for injection were as follows: RAPA solution (10 μmol/L) (HY-10219, MedChemExpress), 3-MA solution (20 μmol/L) (HY-19312, MedChemExpress). miR-542-3p agomir and miRNA agomir negative control (200 nmol/L; Ribobio, Guangzhou, China), miR-542-3p antagomir and miRNA antagomir negative control (200 nmol/L; Ribobio, Guangzhou, China), ATG4D antisense oligonucleotide (ATG4D ASO, 200 nmol/L; Ribobio, Guangzhou, China).

### Immunofluorescence

The TG tissues were removed from experimental mice on day 5 after corneal epithelial debridement, embedded in optimal cutting temperature compound, and stored at − 80 °C. Frozen sections were cut at a thickness of 5 μm. After fixing the sections with 4% paraformaldehyde, the sections were permeabilized and blocked, then incubated with the corresponding primary antibodies and Alexa Fluor-labeled secondary antibodies. Next, the tissues were counterstained with DAPI to visualize the cell nuclei. Finally, the fluorescence intensities were estimated using a fluorescence microscope (Leica, Germany). At least three independent experiments were performed. The primary antibodies used were as follows: anti-p62 antibody (P0067; Sigma-Aldrich, USA), anti-LC3B (ab192890; Abcam, USA), and anti-ATG4D (ab237751; Abcam). The imaging parameters of the same protein were consistent for all experiments (Additional file 1: Table S1). 

### Transmission electron microscopy

TG tissue were removed and cut into 1 mm^3^ sections then immersed in electron microscope fixative and 1% osmic acid. Using gradient ethanol and acetone solutions, the fixed tissues were dehydrated for 15 min. Subsequently, the tissues were embedded in epoxy resins and baked in an oven at 60 °C for 48 h, then cut into ultrathin Sections (80 nm) with an ultramicrotome. Sections were dried after staining with 2% double uranium-lead citrate. Transmission electron microscopic (HT-7700; Hitachi LTD., Japan) images were acquired to determine the number and volume of autophagosomes.

### miRNA sequencing and data analysis

Three mice from each of the control and diabetic groups were collected, and the TG tissue were subjected to miRNA sequencing. Libraries were constructed, and the quality of the amplified libraries was verified with an Agilent 2100 Bioanalyzer (USA). Fifty sequencing cycles were performed using an Illumina NextSeq 500 (Illumina, USA). miRNAs with mean counts per million reads above one in each group were included in the statistical analysis, and paired samples were screened for differentially expressed miRNAs (DEmiRNAs) using the edgeR analytic tool. Only DEmiRNAs with *P* < 0.05, |log2 fold change|≥ 0.585 were included in the subsequent studies (Additional file 1: Tables S2 and S3).

TargetScan and miRDB are databases for miRNA target prediction. We predicted the target genes of miRNAs, from which we screened the target genes with a target score of at least 70. Kyoto Encyclopedia of Genes and Genomes (KEGG) analysis of target genes was conducted to screen unwanted autophagy pathway-related mRNAs, and Cytoscape was used to visualize the animal autophagy pathway-related miRNA-mRNA regulatory network.

### Quantitative real-time polymerase chain reaction (qRT-PCR)

The PrimeScript^™^ First Strand cDNA Synthesis kit (Takara, Japan) and miRNA First Strand cDNA Synthesis kit (Tailing Reaction) (Sangon Biotech, Shanghai, China) were utilized for cDNA synthesis. cDNA was amplified with qRT‒PCR with BlasTaq^™^ 2X qPCR MasterMix (Applied Biological Materials Inc., Canada) on an ABI7500 Real-Time PCR system (Applied Biosystems, Singapore), and the expression levels were analyzed with the 2^−ΔΔCt^ comparison method using β-actin and U6 as internal references. Sangon Biotech (Shanghai) Co., Ltd. designed the primers in this study (Additional file 1: Table S4). At least three independent experiments were performed.

### Dual-luciferase gene reporter assay

To clarity the interaction between mmu-miR-542-3p and ATG4D, plasmid vectors containing wild-type or mutant ATG4D were first constructed (Hanbio, Shanghai, China). Thereafter, the wild-type or mutant ATG4D plasmid was cocultured with cells transfected with miR-542-3p or negative control (NC), and luciferase activity was detected. Three independent experiments were performed.

### Evaluation of ocular surface toxicity

miR-542-3p antagomir is the chemically-modified reverse complementary strand of miR-542-3p: 2 phosphorothioates at the 5’ end, 4 phosphorothioates at the 3’ end, 3’ end cholesterol group, and full-length nucleotide 2’-methoxy modification. We configured miR-542-3p antagomir powders (Ribobio, Guangzhou, China) with sterilized ddH_2_O to 200 nmol/L for subconjunctival injection. Experimental mice were injected with 5 µL solution subconjunctivally every two days for one week. During this period, intraocular pressure (IOP) was measured with a Tonolab tonometer (icare, Shanghai, China) on days 0, 1, 3, 5, and 7 (averaged three times). Corneal transparency and corneal epithelial integrity were evaluated under a slit lamp on day 7. Afterward, corneas were embedded in paraffin and sectioned (thickness 3 µm) for hematoxylin and eosin staining to measure corneal thickness with an optical microscope (Leica, Germany). At least three mice in each group for were used separate independent experiments.

### Statistical analysis

Experiments were conducted at least three times independently. GraphPad Prism v.9.5.1 software (GraphPad Software, Inc., San Diego CA) was used to statistically analyze the data. Data were expressed as the mean ± standard error of the mean (SEM) and were compared between groups using an unpaired t test or one-way analysis of variance (ANOVA). Pearson coefficients were calculated to evaluate the correlation between the two groups of variables. A *P* value < 0.05 was considered statistically significant.

## Results

### Sustained hyperglycemia delayed corneal debridement healing in mice

Compared to age-matched controls, most STZ-treated mice remained in a hyperglycemic state (> 16.7 mmol/L) and had significantly lower body weights (Fig. 1b, c), consistent with the clinical features of diabetes mellitus. Diabetic mice showed significantly reduced corneal sensation than controls, suggesting that mice developed corneal neuropathy in a persistent hyperglycemic state (Fig. 1d). Furthermore, to investigate the impact of diabetic corneal neuropathy on nerve regeneration and epithelial healing, we performed corneal epithelial debridement healing experiments (Fig. 1a). As expected, the corneal nerve density of diabetic mice was significantly lower than that of controls on day 5 after debridement (Fig. 1e). Corneal epithelium healing was significantly delayed at 12, 24, and 36 h (Fig. 1f). Therefore, we confirmed that persistent hyperglycemia led to corneal neuropathy in mice and delayed the healing of corneal epithelial debridement.

### Enhanced autophagy promoted corneal debridement healing in T1DM mice

**Fig. 2:**
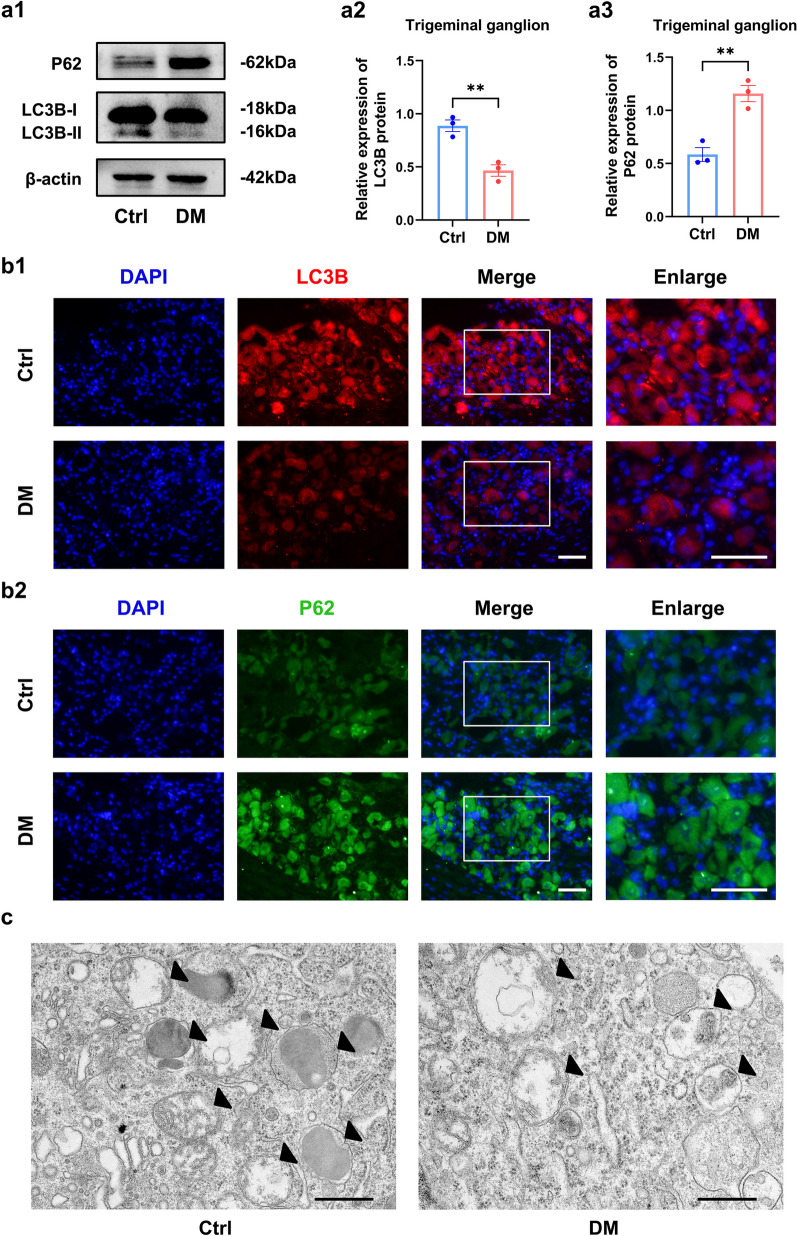
Autophagic flux is restricted in diabetic trigeminal ganglion (TG) tissue. **a** Western blot analysis clarified autophagy proteins expression in TG tissue of normal (Ctrl) and diabetic (DM) mice (n = 3 per group). **a1** Western blot bands of LC3B and P62 proteins. Quantified intensities of Western blot bands for LC3B (**a2**) and P62 (**a3**) compared with β-actin. **b** Immunofluorescence analysis of LC3B (**b1**) and P62 (**b2**) protein expression in TG tissue of Ctrl and DM mice (n = 3 per group). **c** Autophagosomes (black arrows) in TG tissue of Ctrl and DM mice under a transmission electron microscope (n = 3 per group). Scale bar (**b**): 50 μm. Scale bar (**c**): 500 nm. **, *P* < 0.01

To clarify the level of autophagy in the corneal nerve of diabetic mice, we first analyzed the expression levels of autophagy marker proteins P62 and LC3B in TG tissue. Western blot and immunofluorescence analysis revealed a significant upregulation of P62 expression in diabetic mice compared with controls, while LC3B expression was downregulated (Fig. 2a, b and Additional file 1: Fig. S1). Moreover, autophagosomes were significantly reduced in diabetic mice (Fig. 2c). Thus, we demonstrate that autophagy was inhibited in the TG tissue of diabetic mice.

**Fig. 3:**
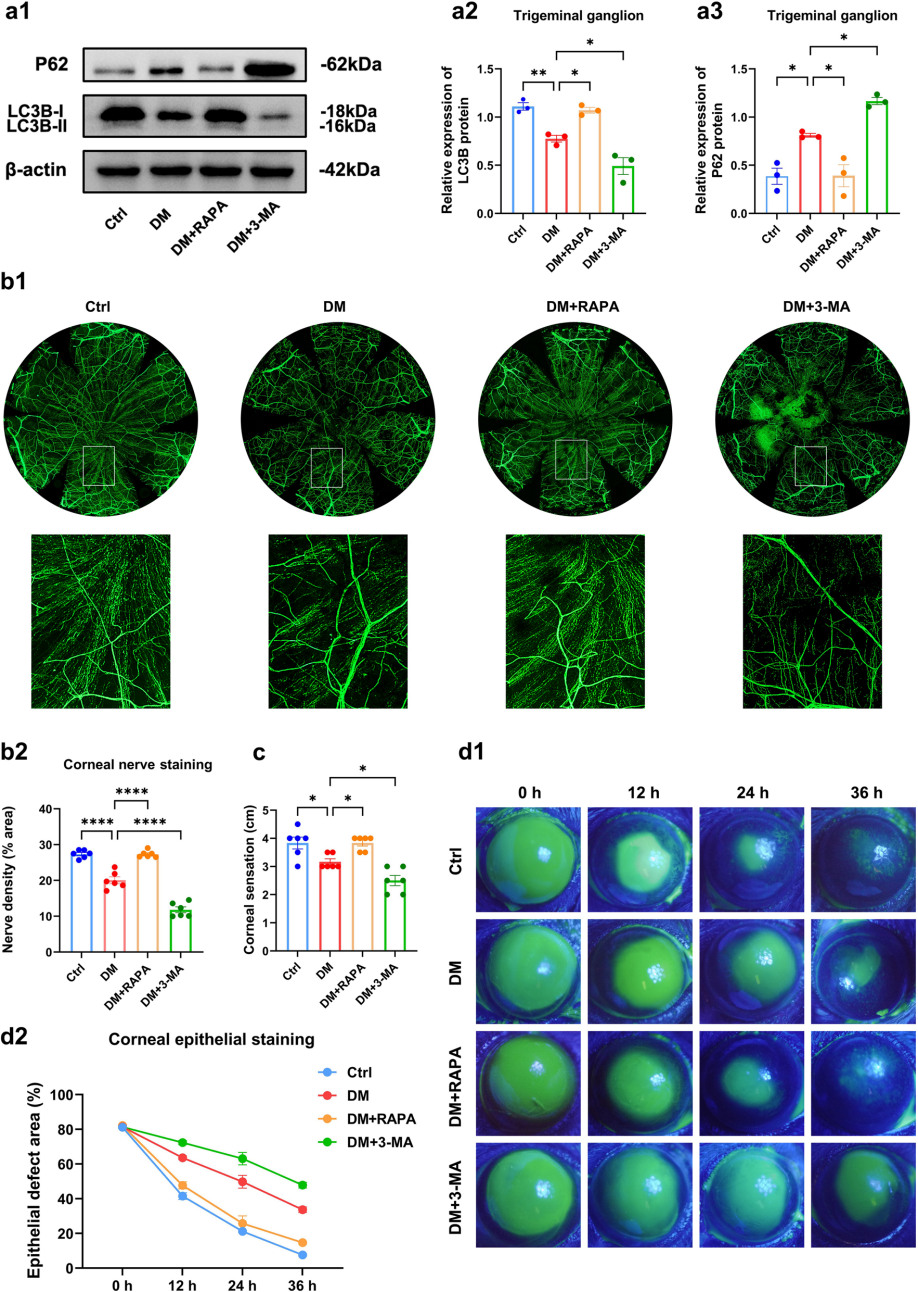
Enhanced autophagy promoted corneal debridement healing in type 1 diabetes mellitus (T1DM) mice. **a** Western blot analysis revealed expression of autophagy proteins in trigeminal ganglion (TG) tissue of control (Ctrl) mice, diabetic (DM) mice, RAPA-treated diabetic mice (DM + RAPA), and 3-MA-treated diabetic mice (DM + 3-MA) (n = 3 per group). **a1** Western blot bands of LC3B and P62 proteins. Quantified intensities of Western blot bands for LC3B (**a2**) and P62 (**a3**) compared with β-actin. **b** Corneal whole-mount staining on day 5 in the Ctrl, DM, DM + RAPA and DM + 3-MA groups after debridement (n = 6 per group). **b1** Fluorescent images of corneal nerve staining. **b2** Peripheral corneal nerve density. **c** Corneal sensation levels of mice on day 5 of each group after debridement (n = 6 per group). **d** Corneas stained with fluorescein sodium of each group at 0, 12, 24, and 36 h after debridement (n = 6 per group). **d1** Fluorescein-stained images of corneas. **d2** Percentage of epithelial defect area. *, *P* < 0.05; **, *P* < 0.01; ****, *P* < 0.0001

Based on the dysregulated autophagy in the diabetic TG tissue, we further analyzed the function of autophagy in diabetic corneal debridement healing. RAPA is commonly used as autophagy agonist and 3-MA is widely used as an autophagy inhibitor. We subconjunctivally injected RAPA or 3-MA and detected autophagy levels in the diabetic TG tissue. The results indicated the downregulation of autophagy protein P62 expression and the upregulation of LC3B expression after RAPA injection, indicating the activation of autophagy in TG tissue (Fig. 3a). In contrast, the autophagy inhibitor 3-MA caused an upregulation of P62 expression as well as a downregulation of LC3B expression, demonstrating the inhibition of autophagy in TG tissue (Fig. 3a). Through corneal debridement experiments, we discovered that RAPA significantly increased corneal nerve density and corneal sensitivity compared to untreated diabetic mice, almost replicating that of controls. In contrast, 3-MA significantly reduced corneal nerve density and corneal sensitivity in diabetic mice (Fig. 3b, c). Consistent with nerve regeneration, RAPA also promoted corneal epithelial healing, whereas 3-MA retarded epithelial healing the diabetic corneas (Fig. 3d). In addition, subconjunctival injection of RAPA and 3-MA did not cause corneal opacity and corneal neovascularization (Additional file 1: Fig. S2). Taken together, these results suggest that activation of autophagy improves epithelial healing and nerve regeneration in diabetic mice.

### Potential autophagy-related miRNA‒mRNA pairs in diabetic TG tissue

**Fig. 4:**
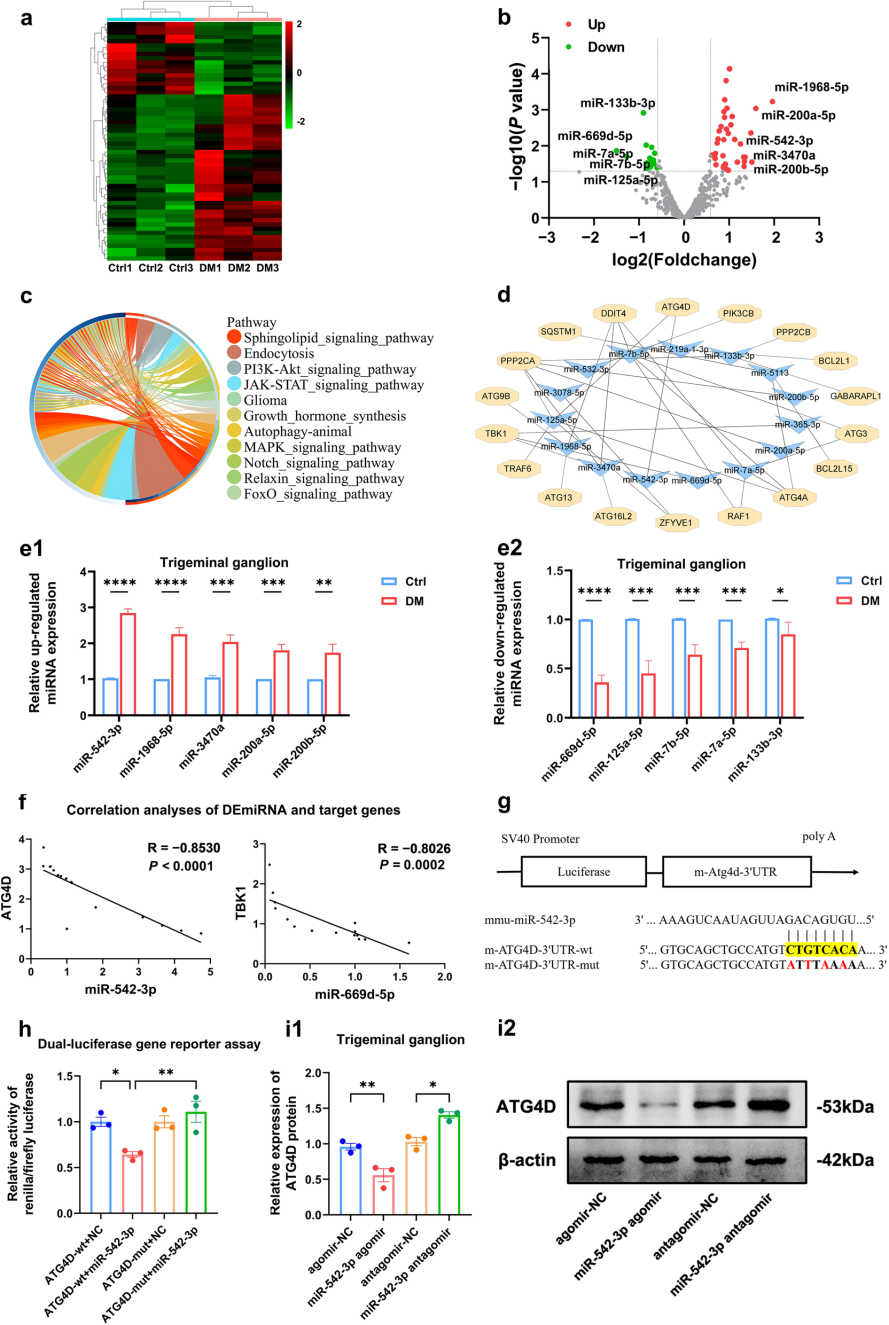
Potential autophagy-related miRNA-mRNA pairs in diabetic trigeminal ganglion (TG) tissue. Heatmap (**a**) and volcano plot (**b**) of DEmiRNAs. **c** Kyoto Encyclopedia of Genes and Genomes enrichment analysis of DEmiRNA target genes. **d** Autophagy-related miRNA-mRNA regulatory network analysis. **e** Quantitative real-time polymerase chain reaction validated the differential expression in TG tissue between control (Ctrl) and diabetic (DM) mice (n = 8). **e1** Differential expression of miR-542-3p, miR-1968-5p, miR-3470a, miR-200a-5p, miR-200b-5p. **e2** Differential expression of miR-669d-5p, miR-125a-5p, miR-7a-5p, miR-7b-5p, and miR-133b-3p. **f** Correlation analyses of miR-542-3p and ATG4D and miR-669d-5p and TBK1 (n = 16). **g** Potential binding site of miR-542-3p to the ATG4D 3' UTR and mutation site of the ATG4D-mut plasmid vector. **h** Dual-luciferase gene reporter assay for relative luciferase activity after cotransfection of ATG4D-wt and ATG4D-mut plasmid vectors with miR-542-3p mimics or negative control in HEK293T cells (n = 3 per group). **i** Western blot analysis showed ATG4D protein expression in TG tissue after subconjunctival injection with miR-542-3p agomir, agomir-NC, miR-542-3p antagomir, and antagomir-NC (n = 3 per group). **i1** Quantified intensities of Western blot bands for ATG4D compared with β-actin. **i2** Western blot bands of LC3B and P62 proteins. *, *P* < 0.05; **, *P* < 0.01; ***, *P* < 0.001; ****, *P* < 0.0001

To identify miRNAs with potential autophagy regulation in diabetic corneal nerves, we obtained differentially expressed miRNAs (DEmiRNAs) in TG tissue samples of control and diabetic mice (three mice each) by miRNA sequencing and screened 56 miRNAs at *P* < 0.05, |log2 fold change|≥ 0.585 (Fig. 4a, b). Subsequently, we predicted the target genes of 56 DEmiRNAs by TargetScan and miRDB to perform KEGG enrichment analysis. Enrichment analysis indicated that the target genes were enriched for animal autophagy as well as PI3K-Akt, MAPK, FOXO1 and other classical signaling pathways associated with autophagy regulation (Fig. 4c).

Based on the above analysis, we identified an autophagy-related DEmiRNA-mRNA interaction network (Fig. 4d). According to the order of |log2 fold change| from more to less in miRNA sequencing, 10 DEmiRNAs that were significantly upregulated (miR-1968-5p, miR-200a-5p, miR-200b-5p, miR-542-3p, miR-3470a) and downregulated (miR-669d-5p, miR-7a-5p, miR-133b-3p, miR-7b-5p, miR-125a-5p) among the autophagy-related DEmiRNA-mRNA interaction network were selected. qRT-PCR verified the expression differences of the 10 DEmiRNAs, and the results supported the sequencing trend overall, in which miR-542-3p and miR-669d-5p exhibited the most significant differences (Fig. 4e and Additional file 1: Figs. S3 and S4). Correlation analysis revealed that miR-542-3p/ATG4D and miR-669d-5p/TBK1 displayed negative correlations, while miR-542-3p showed a stronger correlation with ATG4D (Fig. 4f).

To further demonstrate that ATG4D is a biological target of miR-542-3p, we obtained potential binding sites for ATG4D in the 3'UTR for phylogenetically conserved miR-542-3p by miRDB (Fig. 4g). Wild-type (ATG4D-wt) and mutant (ATG4D-mut) dual-luciferase reporter vectors containing ATG4D 3'UTR sequences were cotransfected with miR-542-3p mimics. The results demonstrate that cotransfection of ATG4D-wt with miR-542-3p mimics exhibited a significant diminution of luciferase activity, but there was no significant difference between ATG4D-mut and control (Fig. 4h). Subsequently, we validated the regulatory role of miR-542-3p on ATG4D in mouse TG tissue. ATG4D protein expression was downregulated in the miR-542-3p agomir group compared with the agomir-NC group, while ATG4D expression was upregulated in the miR-542-3p antagomir group compared with the antagomir-NC group (Fig. 4i). Hence, these results confirm that miR-542-3p regulates ATG4D expression. This led us to hypothesize that miR-542-3p plays a significant regulatory role in autophagy by binding to ATG4D in diabetic TG tissue.

### Suppression of miR-542-3p activated autophagy in diabetic TG tissue

**Fig. 5:**
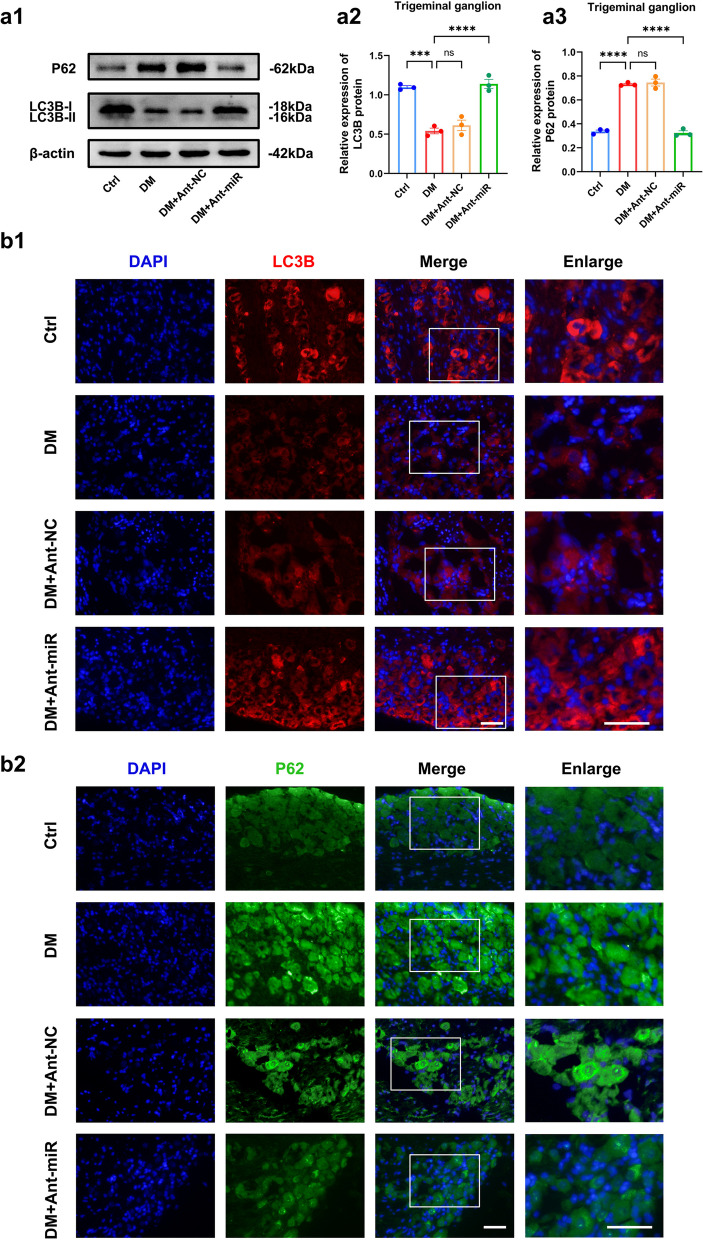
Suppression of miR-542-3p activated autophagy in diabetic trigeminal ganglion (TG) tissue. **a** Western blot analysis of autophagy proteins in TG tissue of control mice (Ctrl), diabetic mice (DM) and diabetic mice injected with antagomir-NC (DM + Ant-NC) or miR-542-3p antagomir (DM + Ant-miR) (n = 3 per group). **a1** Western blot bands of LC3B and P62 proteins. Quantified intensities of Western blot bands for LC3B (**a2**) and P62 (**a3**) compared with β-actin. **b** Immunofluorescence analysis showed LC3B (**b1**) and P62 (**b2**) protein expression in TG tissue of each group. (n = 3 per group). Scale bar: 50 μm. ns, *P* > 0.05; ***, *P* < 0.001; ****, *P* < 0.0001

To explore the influence of miR-542-3p on autophagy in the diabetic corneal nerve, we examined autophagic proteins after subconjunctival injection of miR-542-3p antagomir. Western blotting and immunofluorescence showed that the miR-542-3p antagomir significantly enhanced LC3B expression and decreased P62 protein expression in TG tissue samples compared with samples from untreated diabetic mice, effectively increasing the level of autophagy (Fig. 5a, b). We thus confirmed that suppression of the highly expressed miR-542-3p activated autophagy in TG tissue of diabetic mice.

### ATG4D is a functional target of miR-542-3p for regulating autophagy in diabetic TG tissue

**Fig. 6:**
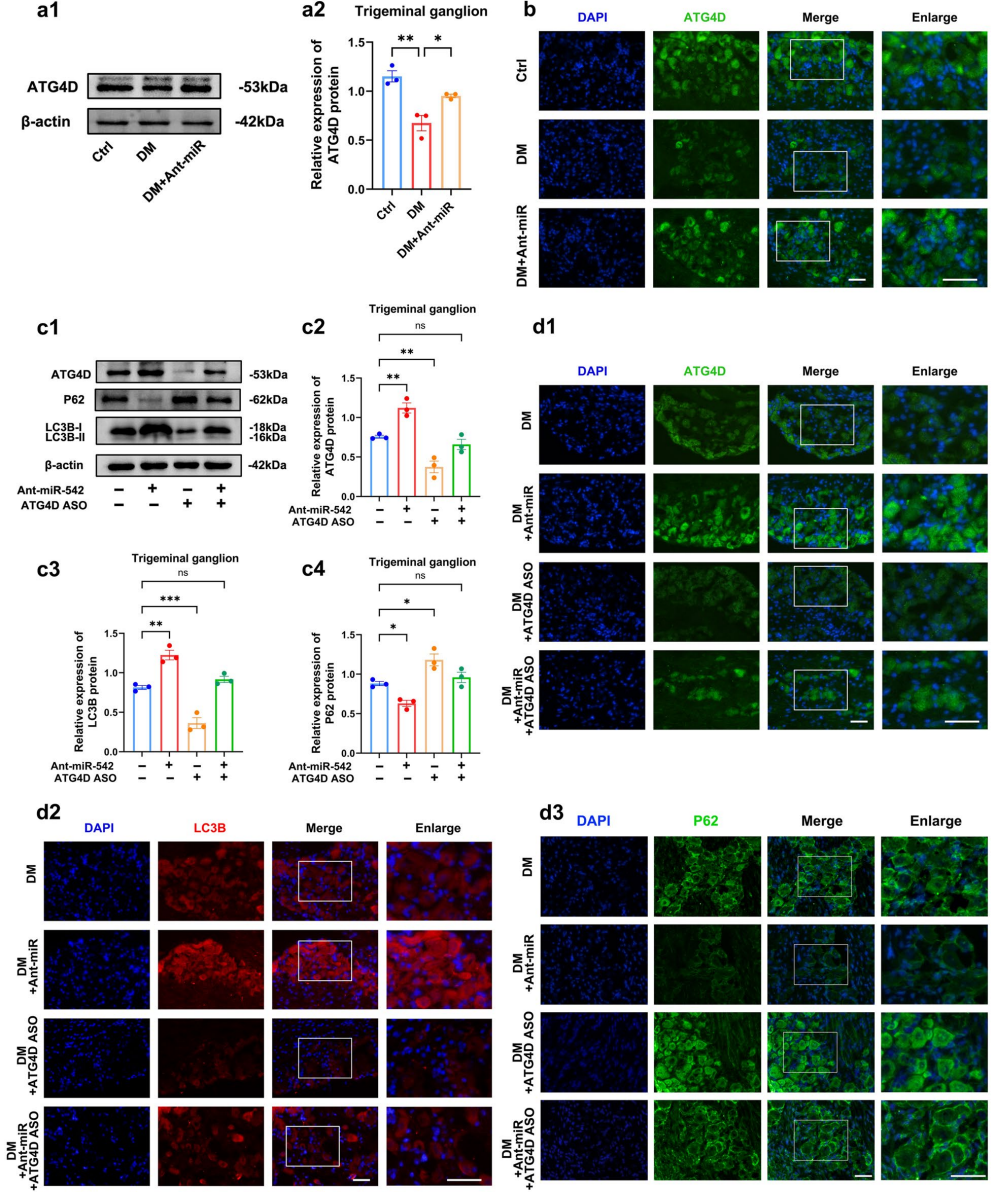
ATG4D is a functional target of miR-542-3p for regulating autophagy in diabetic trigeminal ganglion (TG) tissue.** a** Western blot analysis detected the expression of ATG4D proteins in TG tissue of control mice (Ctrl), diabetic mice (DM), and diabetic mice injected with miR-542-3p antagomir (DM + Ant-miR) (n = 3 per group). **a1** Western blot bands of ATG4D protein. **a2** Quantified intensities of Western blot bands for ATG4D compared with β-actin. **b** Immunofluorescence analysis detected the expression of ATG4D proteins in TG tissue of Ctrl, DM, and DM + Ant-miR (n = 3 per group). **c** Western blot analysis of LC3B, P62, ATG4D proteins in TG tissue of DM, DM + Ant-miR, diabetic mice injected with ATG4D ASO (DM + ATG4D ASO), and diabetic mice injected with miR-542-3p antagomir and ATG4D ASO (DM + Ant-miR + ATG4D ASO) (n = 3 per group). **c1** Western blot bands of LC3B, P62, ATG4D proteins. Quantified intensities of Western blot bands for ATG4D (**c2**), LC3B (**c3**) and P62 (**c4**) compared with β-actin. **d** Immunofluorescence analysis showed the expression of ATG4D (**d1**), LC3B (**d2**) and P62 (**d3**) proteins in TG tissue of DM, DM + Ant-miR, DM + ATG4D ASO, and DM + Ant-miR + ATG4D ASO (n = 3 per group). Scale bar: 50 μm. ns, *P* > 0.05; *, *P* < 0.05; **, *P* < 0.01; ***, *P* < 0.001

ATG4D is a biological target of miR-542-3p. Diabetic mice showed significantly less ATG4D expression in TG tissue than controls, and ATG4D protein expression was significantly upregulated after knockdown of miR-542-3p in diabetic mice, as expected (Fig. 6a, b). To elucidate that miR-542-3p affects autophagy in diabetic TG tissue via ATG4D, we knocked down miR-542-3p while inhibiting ATG4D by subconjunctival injection of ATG4D ASO. ATG4D ASO successfully inhibited the expression of ATG4D protein and antagonized the increase of ATG4D protein expression by miR-542-3p antagomir (Fig. 6c2, d1). Furthermore, ATG4D ASO counteracted the upregulation of LC3B and the P62 downregulation by miR-542-3p antagomir, indicating that ATG4D ASO abolishes the protection of the miR-542-3p antagomir on diabetic TG tissue (Fig. 6c3, c4, and d2, d3). Together, this evidence suggest that ATG4D is a functional target for miR-542-3p-regulated autophagy in diabetic TG tissue.

### miR-542-3p targeting of ATG4D influenced corneal nerve regeneration and epithelial healing in T1DM mice

**Fig. 7:**
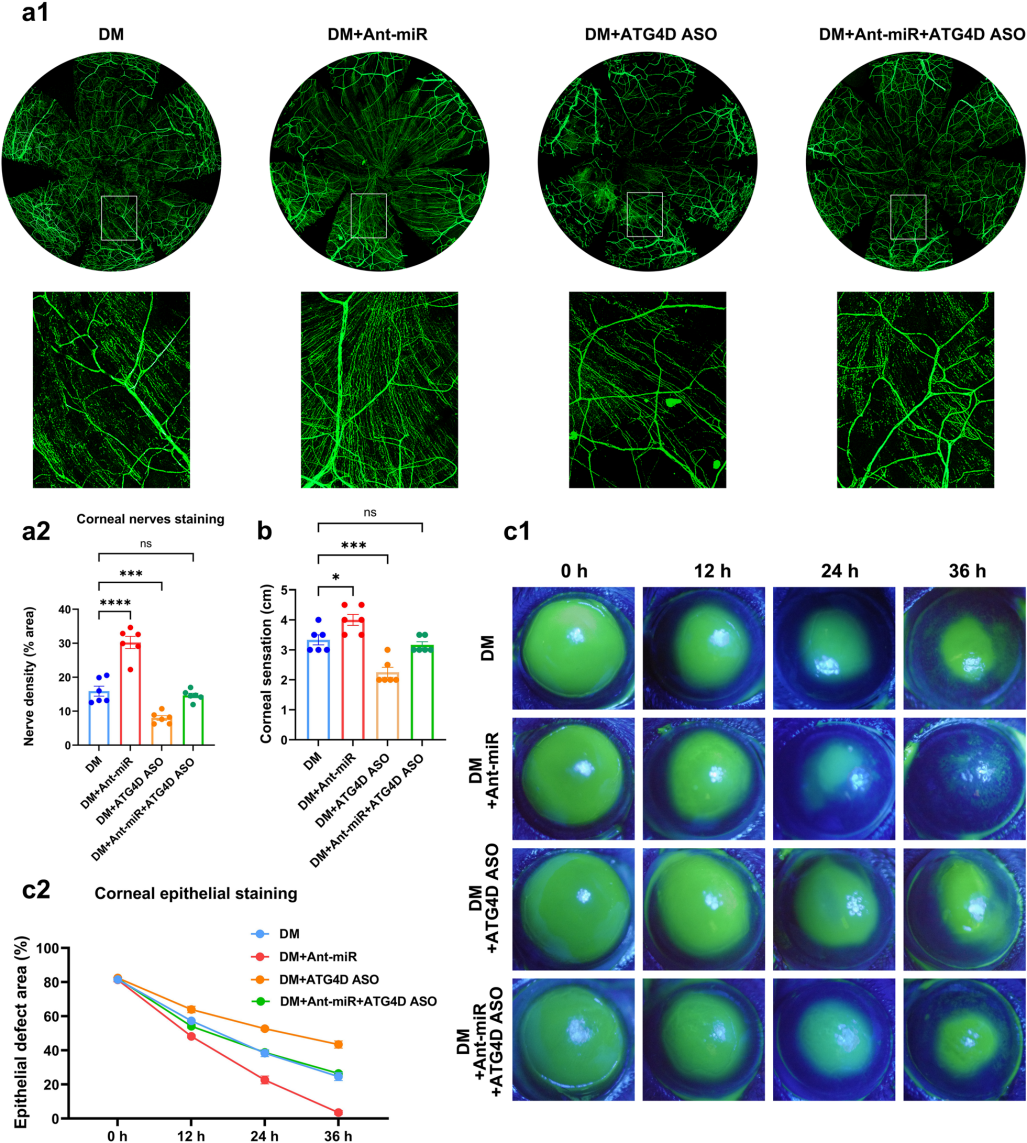
miR-542-3p targeting of ATG4D influenced corneal nerve regeneration and epithelial healing in type 1 diabetes mellitus (T1DM) mice. **a** Corneal nerves whole-mount staining on day 5 of control mice (Ctrl), diabetic mice (DM), diabetic mice injected with miR-542-3p antagomir (DM + Ant-miR), diabetic mice injected with ATG4D ASO (DM + ATG4D ASO), and diabetic mice injected with miR-542-3p antagomir and ATG4D ASO (DM + Ant-miR + ATG4D ASO) after debridement (n = 6 per group). **a1** Fluorescent images of corneal nerve staining. **a2** Peripheral corneal nerve density. **b** Corneal sensation of each group on day 5 after debridement (n = 6 per group). **c** Corneas stained with fluorescein sodium of each group at 0, 12, 24, and 36 h after debridement (n = 6 per group). **c1** Fluorescein-stained images of corneas. **c2** Percentage of epithelial defect area. ns, *P* > 0.05; *, *P* < 0.05; ***, *P* < 0.001; ****, *P* < 0.0001

To clarify the importance of miR-542-3p on diabetic corneal debridement recovery and whether it was dependent on ATG4D, we injected miR-542-3p antagomir and ATG4D-ASO subconjunctivally into diabetic mice to observe epithelial healing and nerve regeneration after corneal injury. The results revealed that miR-542-3p antagomir promoted corneal nerve regeneration and improved corneal sensitivity in diabetic mice. In contrast, ATG4D-ASO hindered corneal nerve regeneration and reduced corneal sensitivity (Fig. 7a, b). Similarly, inhibition of miR-542-3p accelerated corneal epithelial recovery in diabetic mice, whereas knockdown of ATG4D prolonged diabetic corneal epithelial healing (Fig. 7c). ATG4D-ASO antagonized the accelerated healing effect of miR-542-3p antagomir, and corneal sensitivity revealed a similar result (Fig. 7a–c). Taken together, miR-542-3p regulates autophagy through ATG4D to affect nerve regeneration and epithelial repair after diabetic corneal injury.

**Fig. 8:**
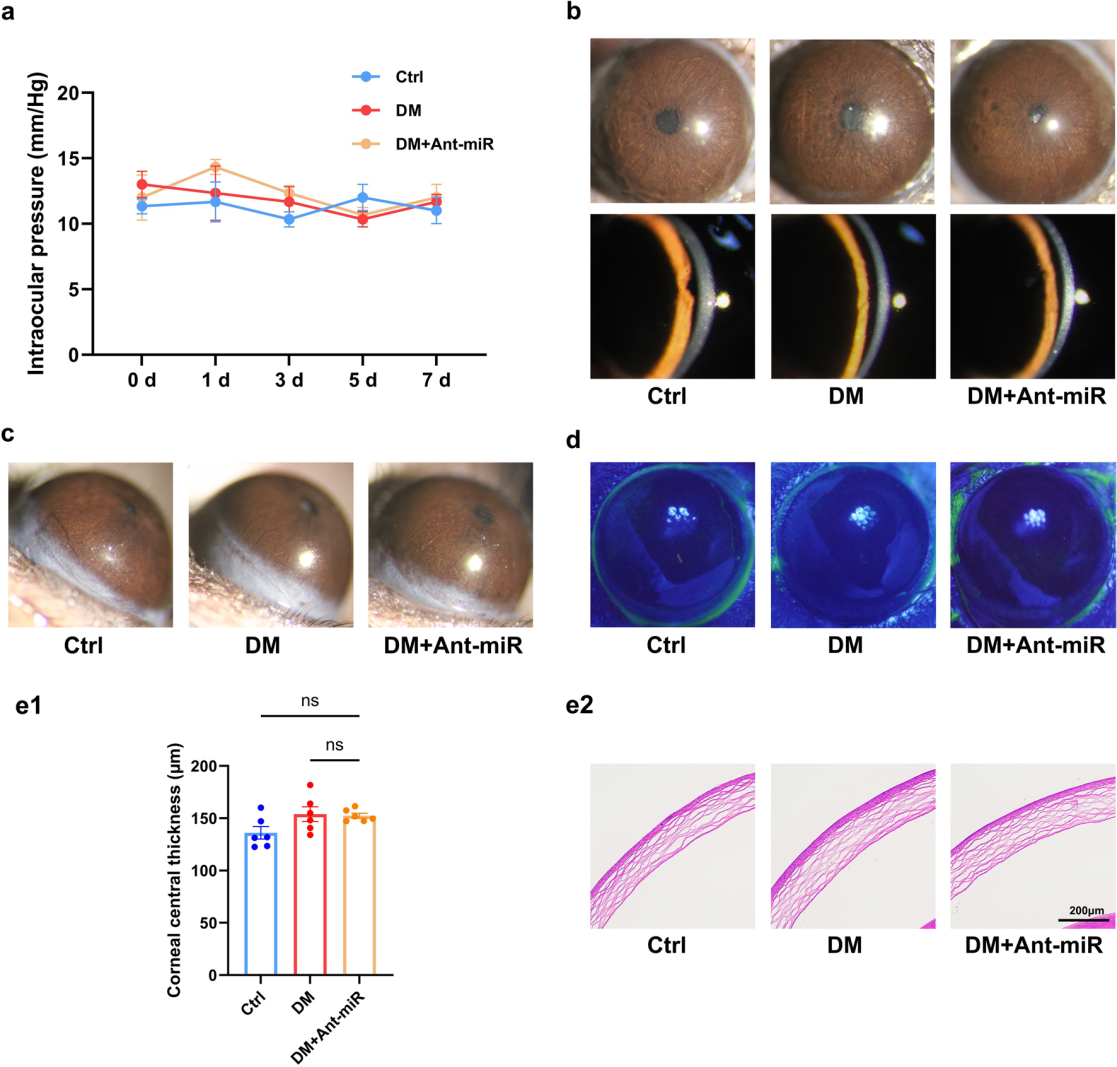
Ocular surface toxicity test of miR-542-3p antagomir by subconjunctival injection. **a** Intraocular pressure of control mice (Ctrl), diabetic mice (DM) and diabetic mice injected with miR-542-3p antagomir (DM + Ant-miR) (n = 6 per group). Corneal transparency (**b**), corneal neovascularization (**c**) and corneal epithelium (**d**) in each group on day 7 (n = 6 per group). **e** Hematoxylin and eosin (HE) staining observation of corneal thickness in each group on day 7 (n = 6 per group). **e1** HE staining of the corneal section. **e2** Measurement of corneal thickness. ns, *P* > 0.05

In addition, we evaluated the safety of miR-542-3p antagomir subconjunctival injection in mice. The IOP, corneal thickness and corneal epithelial integrity of miR-542-3p antagomir-treated mice were similar to those of untreated mice (Fig. 8a, d, e). Meanwhile, no visible corneal opacity and corneal neovascularization were found in miR-542-3p antagomir-treated mice (Fig. 8b, c), confirming its good ocular surface tolerance without obvious toxicities.

## Discussion

**Fig. 9:**
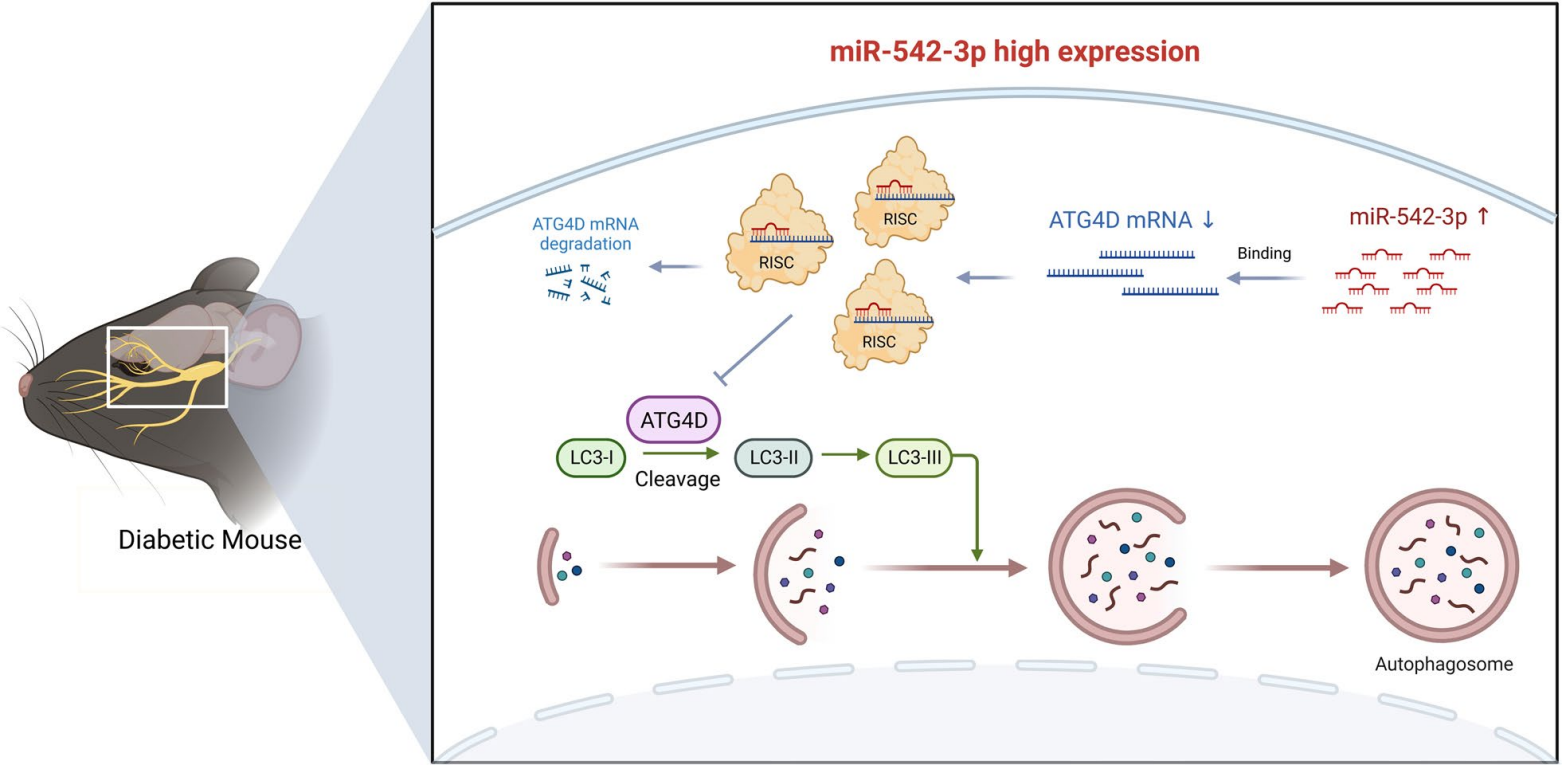
Modes of the miR-542-3p/ATG4D axis in regulating autophagy in diabetic corneal nerve

Corneal neuropathy is currently considered an important cause of DK [5, 8]. The mechanism responsible for diabetic corneal neuropathy is unclear, and effective treatments are limited. In this study, we clarified that the activation of autophagy promoted diabetic corneal debridement healing. We identified miR-542-3p by miRNA sequencing and determined the significance of the miR-542-3p/ATG4D axis in regulating autophagy in corneal epithelial healing and nerve regeneration (Fig. 9). Furthermore, the clinical translational potential of miR-542-3p antagomir was demonstrated and proposed for treating DK.

An increasing number of studies have shown that dysfunctional autophagy leads to neurological dysfunction and neurodegenerative pathologies [32–34]. Aberrant expression of Atg4B and Atg5 has also been found in diabetic corneal nerves, suggesting that dysregulated autophagy is also involved in diabetic corneal neuropathy [21, 22]. Autophagy plays an adaptive role in the cellular stress response, facilitating tissue renewal and tissue repair under moderate activation of autophagy [35–37]. In this study, we clarified the significantly decreased autophagy in the diabetic corneal nerve, which delayed corneal epithelial healing. The autophagy agonist, RAPA, promoted diabetic corneal nerve regeneration and epithelial repair, which provides important evidence for the therapeutic modulation of autophagy to treat corneal injury in patients with diabetes.

The regulatory mechanisms of autophagy are complex and involve both the RNA and protein levels. Emerging studies have reported that a variety of miRNAs operate to directly target autophagy-related signaling proteins to regulate autophagy at the posttranscriptional level [25, 38]. Although the regulatory role of miR-34c and miR-181a-5p in autophagy in the diabetic corneal nerve has been suggested in previous studies [21, 22], the miRNA profile of autophagy regulation in the diabetic corneal nerve has not been well described. In this study, we identified autophagy-regulated miRNA-mRNA networks, which greatly helps in dissecting the complex autophagy regulatory mechanism of DK. Our study revealed that among the regulatory networks, miR-542-3p exerts a significant role in the diabetic TG tissue. Thus, miR-542-3p may serve as a potential biomarker and treatment option for diabetic corneal neuropathy going forward. The specific roles of the other miRNA-mRNAs in the autophagy regulatory network in DK need to be further explored.

Our study identified ATG4D, an important protein for autophagosome membrane formation, as a key target protein of miR-542-3p [39]. ATG4D is the main delipidating enzyme of mATG8 and facilitates autophagosome formation, removing abnormal protein accumulation to protect neurons [40–42]. Deficiency or mutation of ATG4D causes cerebellar neurodegeneration and neurodevelopmental disorders [34, 41]. However, there are no studies on ATG4D in diabetic corneal neuropathy. For the first time, we used the ASO technique to inhibit ATG4D and clarified the role of ATG4D in aiding corneal recovery in diabetic mice via autophagy. Additionally, our findings strongly indicate that miR-542-3p targets ATG4D to regulate autophagy in diabetic corneal nerve, thereby affecting corneal nerve regeneration and epithelial repair.

Some ocular preparations in the treatment of ocular diseases also possess ocular surface toxic effects such as increased IOP, damage to the corneal epithelium, induced ocular surface inflammation, corneal edema and so on [43–45]. Therefore, we assessed the ocular surface safety of miR-542-3p antagomir to gain a more comprehensive understanding of its potential benefits and risks to provide additional evidence for its clinical application. In our study, we used sterile ddH_2_O to configure chemically-modified miR-542-3p antagomir for subconjunctival injection. The results confirmed that miR-542-3p antagomir performed well in promoting corneal epithelial recovery and nerve regrowth without obvious toxic effects including high IOP, corneal edema, corneal opacity, corneal neovascularization, and corneal epithelial damage. Based on the identified therapeutic target miR-542-3p, we proposed the clinical translational potential of miR-542-3p antagomir as a treatment for DK for the first time. Notably, activating autophagy by RAPA also showed effects, but extensive intervention of autophagy may affect the physiological function of normal tissues. For the consideration of therapeutic target specificity, we prefer the more specific intervention of miR-542-3p antagomir. However, pharmaceutical dynamics of miR-542-3p antagomir need further investigation, and development into an easy-to-use eye drop would be a more practical approach (Additional file 1).

## Conclusion

Taken together, we demonstrated that dysregulation of autophagy is a significant factor in delayed healing after diabetic corneal injury. miR-542-3p is a potential biomarker for diabetic corneal neuropathy. Inhibition of miR-542-3p augments autophagy to promote corneal debridement healing, providing a new therapeutic choice for clinical treatment.

### Supplementary Information

**Additional file 1: Figure S1.** Immunofluorescence analysis of LC3B and P62 proteins with co-localized staining of nerves in corneal of control mice and diabetic mice. (n = 3 per group). Scale bar: 50 μm. **Figure S2**. Corneal opacity and corneal neovascularization of diabetic mice injected with RAPA (DM + RAPA) and diabetic mice injected with 3-MA (DM + 3-MA). n = 6 per group. **Figure S3.** Quantitative real-time polymerase chain reaction (qRT-PCR) validated the expression of miR-542-3p between corneal and trigeminal ganglion (TG) tissues. n = 5 per group. Ctrl, control mice; DM, diabetic mice; ***, *P* < 0.001. **Figure S4.** Quantitative real-time polymerase chain reaction (qRT-PCR) validated the differential expression of miR-542-3p tissue between control (Ctrl) and diabetic (DM) mice at 0, 12, 24, 48, 72 h after debridement (n = 3 per group). *, *P* < 0.05; **, *P* < 0.01. **Table S1.** Image acquisition parameters for immunofluorescence. **Table S2.** Upregulated miRNA found from RNA-seq. **Table S3.** Downregulated miRNA found from RNA-seq. **Table S4.** Primer sequences and conditions for conventional Quantitative real-time polymerase chain reaction (qRT-PCR). 

## Data Availability

All data are included in the manuscript and supplementary materials. The resources during the current study are available upon reasonable request.
