# Supplementary material for: Inhibition of miR-542-3p augments autophagy to promote diabetic corneal wound healing
Source: Eye Vis (Lond). 2024 Jan 3;11:3. doi: 10.1186/s40662-023-00370-1 (PMC10763460; doi:10.1186/s40662-023-00370-1)
Supplement: Supplementary file 1 — Additional file 1: Figure S1. Immunofluorescence analysis of LC3B and P62 proteins with co-localized staining of nerves in corneal of control mice and diabetic mice. (n = 3 per group). Scale bar: 50 μm. Figure S2. Corneal opacity and corneal neovascularization of diabetic mice injected with RAPA (DM + RAPA) and diabetic mice injected with 3-MA (DM + 3-MA). n = 6 per group. Figure S3. Quantitative real-time polymerase chain reaction (qRT-PCR) validated the expression of miR-542-3p between corneal and trigeminal ganglion (TG) tissues. n = 5 per group. Ctrl, control mice; DM, diabetic mice; ***, P < 0.001. Figure S4. Quantitative real-time polymerase chain reaction (qRT-PCR) validated the differential expression of miR-542-3p tissue between control (Ctrl) and diabetic (DM) mice at 0, 12, 24, 48, 72 h after debridement (n = 3 per group). *, P < 0.05; **, P < 0.01. Table S1. Image acquisition parameters for immunofluorescence. Table S2. Upregulated miRNA found from RNA-seq. Table S3. Downregulated miRNA found from RNA-seq. Table S4. Primer sequences and conditions for conventional Quantitative real-time polymerase chain reaction (qRT-PCR). [file 40662_2023_370_MOESM1_ESM.docx]

Additional Figure S1. Immunofluorescence analysis of LC3B and P62 proteins with co-localized staining of nerves in corneal of control mice and diabetic mice. (n = 3 per group). Scale bar: 50 μm.


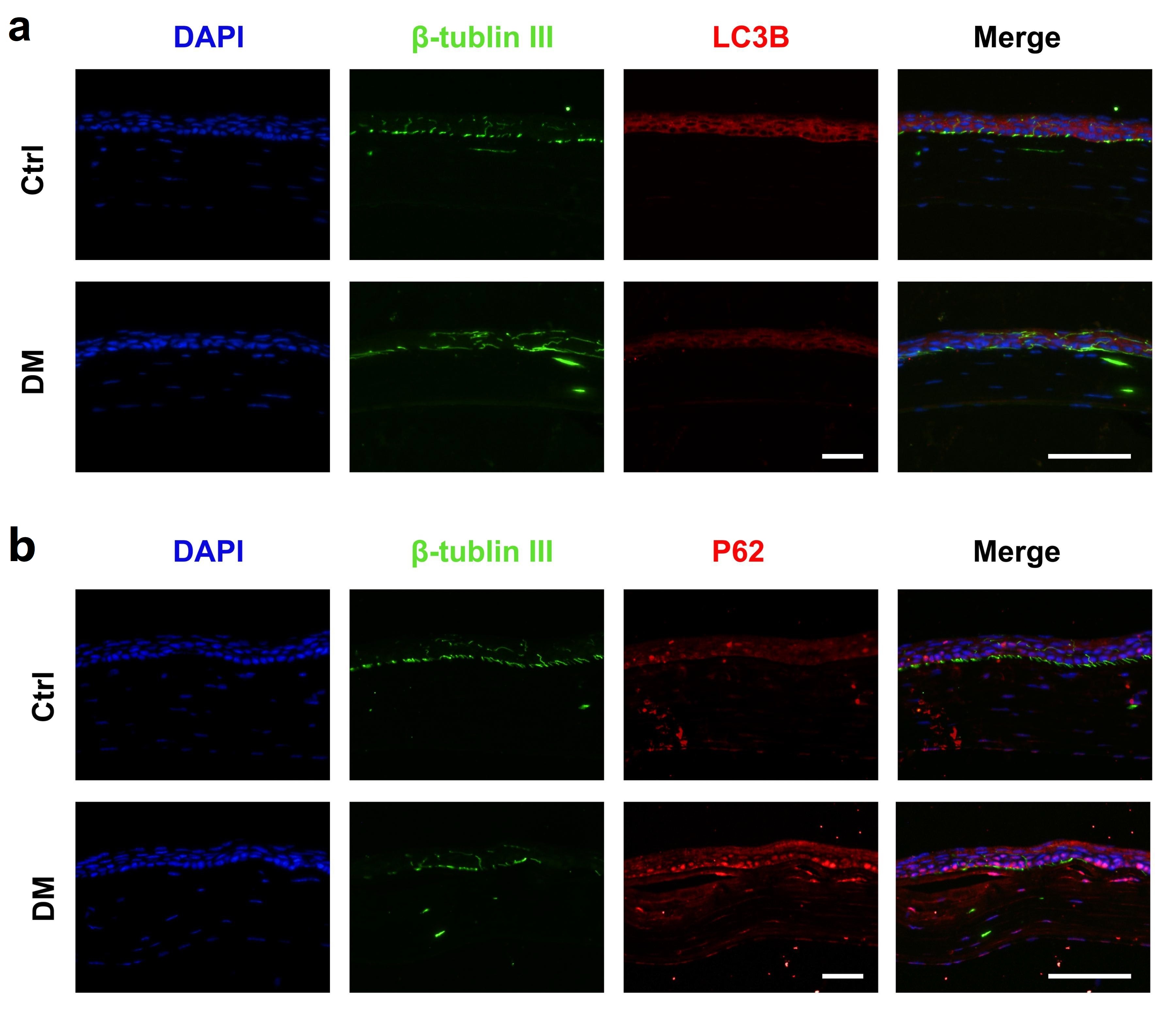


Additional Figure S2. Corneal opacity and corneal neovascularization of diabetic mice injected with RAPA (DM+RAPA) and diabetic mice injected with 3-MA (DM+3-MA). n=6 per group.


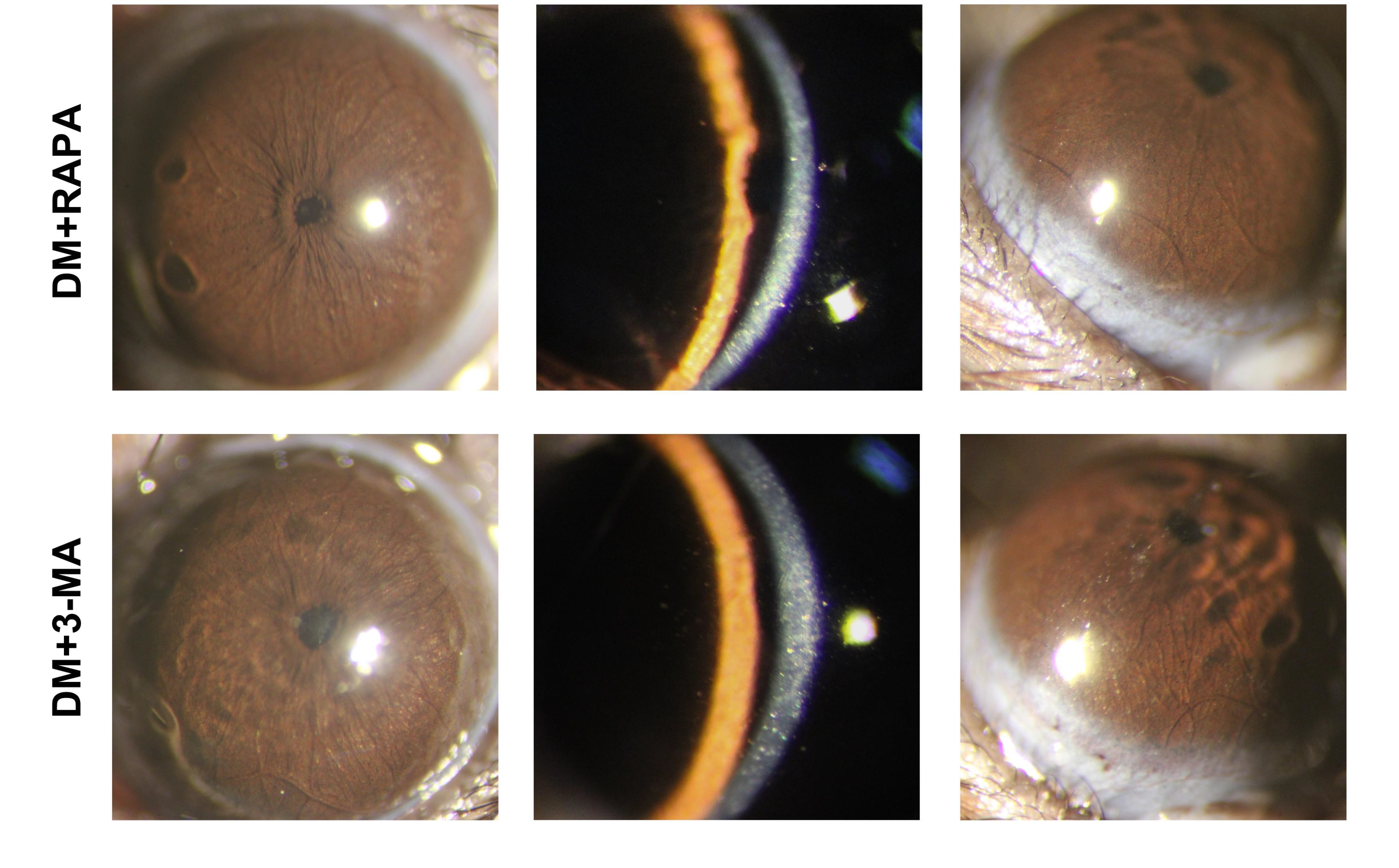


Additional Figure S3. Quantitative real-time polymerase chain reaction (qRT-PCR) validated the expression of miR-542-3p between corneal and trigeminal ganglion (TG) tissues. n = 5 per group. Ctrl, control mice; DM, diabetic mice; ***, *P* < 0.001.


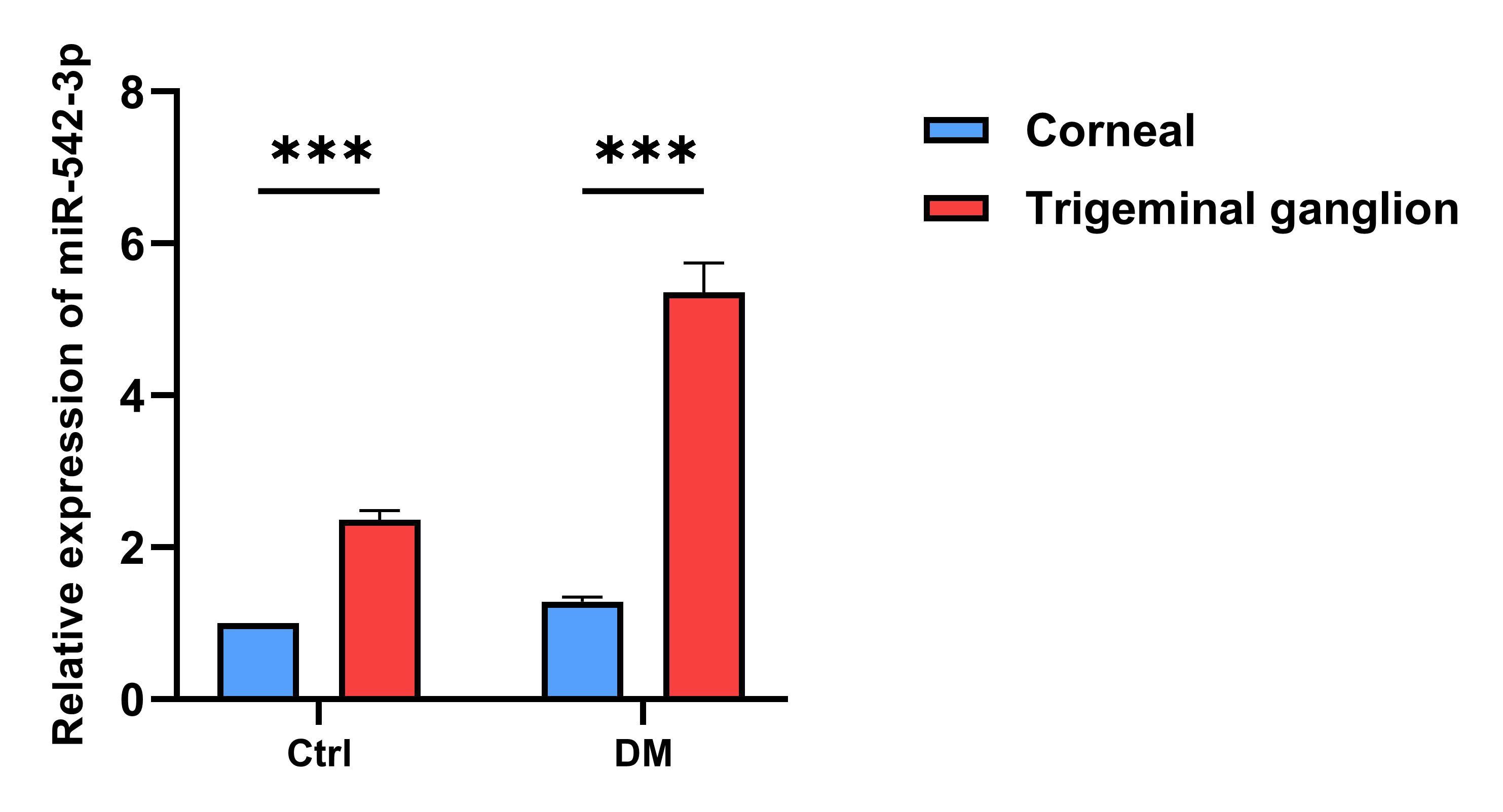


Additional Figure S4. Quantitative real-time polymerase chain reaction (qRT-PCR) validated the differential expression of miR-542-3p tissue between control (Ctrl) and diabetic (DM) mice at 0, 12, 24, 48, 72 hours after debridement (n = 3 per group). *, *P* < 0.05; **, *P* < 0.01.


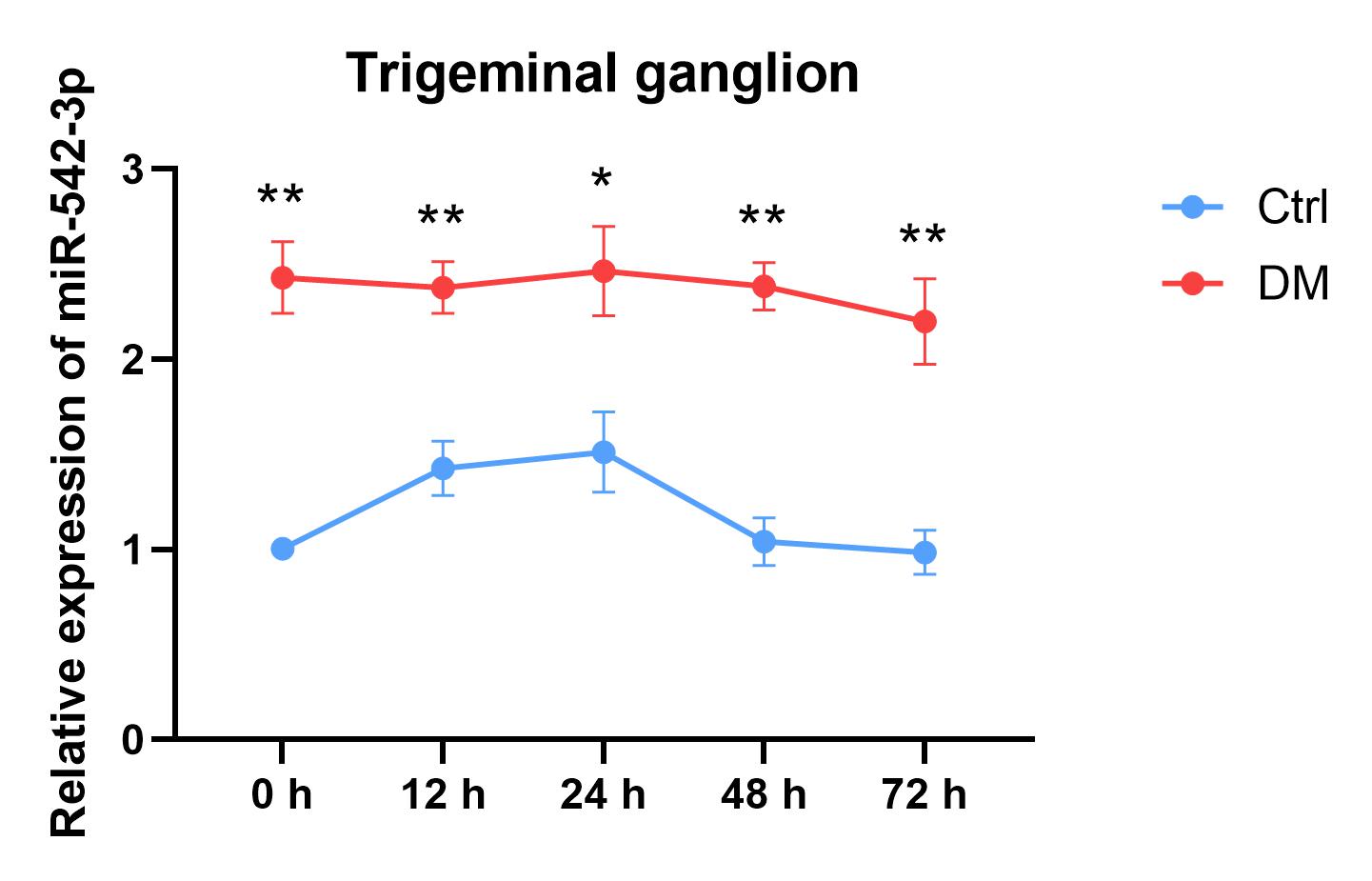


**Additional Table S1. Image acquisition parameters for immunofluorescence.**

| Type* | Exposure time (ms) | Gain |
| --- | --- | --- |
| DAPI | 50 | 1 |
| LC3B | 300 | 1 |
| P62 | 300 | 1 |
| ATG4D | 800 | 1 |

*The imaging parameters of the same protein were consistent for all experiments.

**Additional Table 2. Upregulated miRNA found from RNA-seq.**

| Mature ID | log2 fold change | Fold change | *P* value |
| --- | --- | --- | --- |
| mmu-miR-1968-5p | 1.962521892 | 3.897426705 | 0.0005846 |
| mmu-miR-200a-5p | 1.591795371 | 3.014242254 | 0.00090985 |
| mmu-miR-341-5p | 1.506617308 | 2.841430264 | 0.028160967 |
| mmu-miR-144-3p | 1.481789772 | 2.79295005 | 0.004340563 |
| mmu-miR-653-5p | 1.355634076 | 2.559095659 | 0.020350034 |
| mmu-miR-200b-5p | 1.337685683 | 2.527455486 | 0.027097065 |
| mmu-miR-451a | 1.33297195 | 2.519210979 | 0.037197988 |
| mmu-miR-3470a | 1.321473789 | 2.499212871 | 0.020419835 |
| mmu-miR-542-3p | 1.251662939 | 2.381157316 | 0.008748416 |
| mmu-miR-5113 | 1.18033766 | 2.266298131 | 0.028070044 |
| mmu-miR-200b-3p | 1.120486046 | 2.174202094 | 0.006586934 |
| mmu-miR-1298-5p | 1.069714281 | 2.099017625 | 0.001522738 |
| mmu-miR-215-5p | 1.038243005 | 2.053724988 | 0.00260157 |
| mmu-miR-146a-5p | 1.007769361 | 2.010799675 | 7.28126E-05 |
| mmu-miR-3095-3p | 0.981641073 | 1.974710376 | 0.004476503 |
| mmu-miR-34c-5p | 0.980547022 | 1.973213444 | 0.047440853 |
| mmu-miR-3964 | 0.955463676 | 1.939202785 | 0.045311952 |
| mmu-miR-378c | 0.951280947 | 1.9335887 | 0.000906916 |
| mmu-miR-142a-3p | 0.940147906 | 1.918724938 | 0.00343416 |
| mmu-miR-31-5p | 0.932528664 | 1.908618371 | 0.000154086 |

**Additional Table 3. Downregulated miRNA found from RNA-seq.**

| Mature ID | log2 fold change | Fold change | *P* value |
| --- | --- | --- | --- |
| mmu-miR-669d-5p | −1.500813474 | 0.353354093 | 0.013713362 |
| mmu-miR-7a-5p | −1.274908791 | 0.413251285 | 0.01956453 |
| mmu-miR-133b-3p | −0.901515612 | 0.535324055 | 0.001217398 |
| mmu-miR-532-3p | −0.841622231 | 0.558015758 | 0.009480529 |
| mmu-miR-219a-1-3p | −0.829305011 | 0.562800295 | 0.029725997 |
| mmu-miR-7b-5p | −0.825363583 | 0.564339961 | 0.041220582 |
| mmu-miR-3078-5p | −0.79778446 | 0.57523188 | 0.032908329 |
| mmu-miR-365-3p | −0.760254993 | 0.590391971 | 0.021773915 |
| mmu-miR-125a-5p | −0.747973202 | 0.595439485 | 0.038052063 |
| mmu-miR-672-5p | −0.737772946 | 0.599664326 | 0.023443419 |
| mmu-miR-335-3p | −0.721947007 | 0.606278679 | 0.010929583 |
| mmu-miR-669c-5p | −0.719610414 | 0.607261405 | 0.025899378 |
| mmu-miR-301a-5p | −0.692123979 | 0.618941954 | 0.024990502 |
| mmu-miR-199b-5p | −0.682363276 | 0.623143667 | 0.024859515 |
| mmu-miR-493-5p | −0.667425341 | 0.629629333 | 0.031179776 |
| mmu-miR-133a-3p | −0.656540766 | 0.634397607 | 0.015970529 |
| mmu-miR-574-5p | −0.602204532 | 0.658746578 | 0.040416816 |

**Additional Table S4. Primer sequences and conditions for conventional Quantitative real-time polymerase chain reaction (qRT-PCR).**

| **Gene** | **Forward primer (5′-3′)** | **Reverse primer (5′-3′)** |
| --- | --- | --- |
| ATG4D | TAAGTTCTGCCAACTCTTCCTT | CGGAGCTGTAGAGTTTCTTCTT |
| TBK1 | CAGTCTTCAGGACATCAGCAGCAG | CTGTCTCTTGGATGCGTGCCTTC |
| β-actin | GTACCACCATGTACCCAGGC | AACGCAGCTCAGTAACAGTCC |
| miR-542-3p | GCCGTGTGACAGATTGATAACTGA | AACGCTTCACGAATTTGCGT |
| miR-1968-5p | TGCAGCTGTTAAGGATGGTGGACT | AACGCTTCACGAATTTGCGT |
| miR-3470a | TCACTTTGTAGACCAGGCTGG | AACGCTTCACGAATTTGCGT |
| miR-200a-5p | CATCTTACCGGACAGTGCTGGA | AACGCTTCACGAATTTGCGT |
| miR-200b-5p | CATCTTACTGGGCAGCATTGGA | AACGCTTCACGAATTTGCGT |
| miR-669d-5p | GCCACTTGTGTGTGCATGTATATGT | AACGCTTCACGAATTTGCGT |
| miR-125a-5p | TCCCTGAGACCCTTTAACCTGTGA | AACGCTTCACGAATTTGCGT |
| miR-7a-5p | GCCTGGAAGACTAGTGATTTTGTTGT | AACGCTTCACGAATTTGCGT |
| miR-7b-5p | GCGGTGGAAGACTTGTGATTTTGTT | AACGCTTCACGAATTTGCGT |
| miR-133b-3p | TTTGGTCCCCTTCAACCAGCTA | AACGCTTCACGAATTTGCGT |
| U6 | CCTGCTTCGGCAGCACA | AACGCTTCACGAATTTGCGT |
